# Special Considerations for Women of Reproductive Age on Anticoagulation

**DOI:** 10.1007/s11606-022-07528-y

**Published:** 2022-05-31

**Authors:** Tali Azenkot, Eleanor Bimla Schwarz

**Affiliations:** 1 Department of Internal Medicine, University of California Davis School of Medicine, 4150 V St, Sacramento, CA 95817 USA; 2 Department of Internal Medicine, Division of General Internal Medicine, University of California San Francisco, San Francisco, CA USA

**Keywords:** anticoagulation, women of reproductive age, menstruation, contraception

## Abstract

Anticoagulation poses unique challenges for women of reproductive age. Clinicians prescribing anticoagulants must counsel patients on issues ranging from menstruation and the possibility of developing a hemorrhagic ovarian cyst to teratogenic risks and safety with breastfeeding. Abnormal uterine bleeding affects up to 70% of young women who are treated with anticoagulation. As such, thoughtful clinical guidance is required to avoid having young women who are troubled by their menses, dose reduce, or prematurely discontinue their anticoagulation, leaving them at increased risk of recurrent thrombosis. Informed by a review of the medical literature, we present current recommendations for assisting patients requiring anticoagulation with menstrual management, prevention of hemorrhagic ovarian cysts, and avoiding unintended pregnancy. The subdermal implant may be considered a first-line option for those requiring anticoagulation, given its superior contraceptive effectiveness and ability to reliably reduce risk of hemorrhagic ovarian cysts. All progestin-only formulations—such as the subdermal implant, intrauterine device, injection, or pills—are generally preferred over combined hormonal pills, patch, or ring. Tranexamic acid, and in rare cases endometrial ablation, may also be useful in managing menorrhagia and dysmenorrhea. During pregnancy, enoxaparin remains the preferred anticoagulant and warfarin is contraindicated. Breastfeeding women may use warfarin, but direct oral anticoagulants are not recommended given their limited safety data. This practical guide for clinicians is designed to inform discussions of risks and benefits of anticoagulation therapy for women of reproductive age.

**Supplementary Information:**

The online version contains supplementary material available at 10.1007/s11606-022-07528-y.

A wide range of women of reproductive age may need to be treated with anticoagulant medications. In addition to those with genetic thrombophilia, women with rheumatologic conditions, vascular disease, and mechanical heart valves and those who have experienced a thrombosis warrant treatment with anticoagulants. Women of reproductive age face unique health challenges related to therapeutic anticoagulation. For example, abnormal uterine bleeding affects up to 70% of menstruating women on oral anticoagulation, compared to up to 33% of young women who are not on anticoagulation^1,2^. Women who ovulate while on anticoagulation also face an increased risk of hemorrhagic ovarian cysts, which can be life threatening and have been reported to affect 1% of young women on anticoagulation.^3,4^ Effective preconception and contraceptive counseling are critical for this high-risk patient population given that pregnancy and the postpartum period increase risk of thrombosis and some anticoagulants are contraindicated during pregnancy or breastfeeding.

Many clinicians have received limited training on the special considerations involved in caring for women of reproductive age.^5,6^ Several resources are available to guide clinician assessment of patient sexual history, reproductive goals, and potential benefit from contraception; however, these tools are not specific to women on anticoagulation.^7–9^ To address this gap, this review summarizes current literature relevant when caring for young women who require treatment with anticoagulation. We also detail topics to ensure effective shared decision-making between young women and the clinical team, with suggested language shown in Table 1.

**Table 1:** Counseling Women of Childbearing Age About Anticoagulation

Topics to cover	Model language
Assess for menstrual concerns	• How do your menstrual cycles tend to affect your life? • Are you troubled by cramping or excess bleeding with your periods? • Have your periods been more of a problem since starting anticoagulation?
Discuss options for menstrual and ovarian suppression	• There are a number of hormonal treatments we can use to help manage your periods.
Advise of risks of using NSAIDs for dysmenorrhea	• Medications such as ibuprofen, which you may have previously used for cramps, will put you at risk for bleeding complications while you are on a blood thinner. • Other options for managing cramps include heating pads and acetaminophen.
Evaluate sexual history Further guidelines available^7–9^:	• What is/are the gender(s) of your current sexual partner(s)?
Assess reproductive goals	• Do you think there’s any chance you are currently pregnant? • How would you feel if you were to become pregnant at this time? • When, if ever, would you like to become pregnant? • Can you tell me a bit about any prior pregnancies? (if recent birth, are you currently breastfeeding?)
Counsel on teratogenic risks	• I need you to know that taking this medication during pregnancy will increase the risk of birth defects.
Discuss contraceptive options	• Tell me about what types of contraception you have used in the past. • There are many ways to safely prevent pregnancy while taking a blood thinner. I’d like you to select the contraceptive that you think will be best for you at this point in your life, recognizing you can always try another option if it is not working out for you.

Throughout this review, the term women is used for all persons with a uterus and ovaries. We recognize that gender identity varies. The unique needs of transgender and post-menopausal women, who may require particular attention to drug-drug interactions and thrombotic risks while on hormone therapy, are detailed elsewhere.^10,11^

## METHODS

To understand rates of abnormal uterine bleeding (AUB) experienced by women of reproductive age on anticoagulation, we searched PubMed and EMBASE using the following key terms: “reproductive health” OR “childbearing age*” OR “reproductive age*” OR “menstrual cycle” OR menstruat* OR menstrual* OR “heavy menstrual bleeding” OR “Menstruation Disturbances” OR “uterine hemorrhage” OR “vaginal bleeding” OR menorrhagia OR “breakthrough bleeding” OR “spotting” OR “dysfunctional uterine bleeding” OR “ovarian cysts” OR “ovarian cyst*” AND (Anticoagulants OR anticoagulant* OR anticoagulat*). We selected articles that included observational and comparative studies, clinical trials, meta-analyses, reviews, and practice guidelines. With the goal of identifying current literature, search dates were restricted to the prior decade (2011–2021), which aligns with the initial US Food and Drug Administration approval of the direct-acting oral anticoagulants. From 204 records screened, 20 articles were included in this review (see Supplementary Material).

We also draw on the IBM Micromedex and LactMed databases for clinical data regarding drug safety and adverse events of six commonly used anticoagulants: apixaban, rivaroxaban, edoxaban, dabigatran, enoxaparin, and warfarin.^12,13^ We will refer to both the anti-Xa inhibitors (i.e., apixaban, rivaroxaban, edoxaban) and direct thrombin inhibitors (i.e., dabigatran) as direct-acting oral anticoagulants (DOACs). By summarizing key information from these resources and select society guidelines, we have created a practical reference to guide clinical discussions with women of reproductive age regarding risks and benefits of anticoagulation.

## ABNORMAL UTERINE BLEEDING EXPERIENCED BY REPRODUCTIVE AGE WOMEN ON ANTICOAGULATION

Abnormal uterine bleeding (AUB) includes heavy menstrual bleeding and bleeding at times outside the usual menstrual cycle (i.e., intermenstrual, postcoital). Heavy menstrual bleeding is menstrual blood loss that interferes with a women’s physical, social, emotional, or material quality of life, or quantified as loss of >80mL menstrual blood loss per cycle in research.^14^ Clinical signs of such bleeding include changing a pad or tampon more than hourly, leaking or soaking through clothing, having to change pads or tampons overnight, menses lasting more than 7 days, and passing clots greater than 2.8 cm.^15^ This can result in fatigue, shortness of breath, or lightheadedness as a result of anemia. When poorly controlled, abnormal uterine bleeding can interfere with work responsibilities and has significant adverse effects on quality of life.^16^

For women on oral anticoagulation, rates of uterine bleeding related to anticoagulant medications in pharmaceutical event reports have been estimated at 2 to 4%.^10,17^ However, this likely underestimates the frequency of troublesome AUB given few women of reproductive age were included in these studies and menstrual history was not collected in a standardized fashion.^18^ Observational studies report higher rates of AUB for women on DOACs ranging from 15.8 to 50%, and up to 66% for women on warfarin.^1,10,18–20^ These studies have indicated that dabigatran may be the preferred anticoagulant for menstruating women, as it has demonstrated lower rates of AUB than warfarin when used by this population.^21–24^ This is hypothesized to result from the high concentration of thrombin in the endometrium, where available thrombin may exceed dabigatran-induced thrombin inhibition to decrease menstrual bleeding.^25^ Rivaroxaban has, in contrast, repeatedly demonstrated worse AUB than apixaban, edoxaban, dabigatran, or warfarin, including with severe uterine bleeding requiring blood transfusion or surgical management.^26^ Rates of AUB with use of common anticoagulants are compared in Table 2.

**Table 2:** Anticoagulant Medication Effects for Women of Reproductive Age

	Warfarin	Enoxaparin	Dabigatran	Rivaroxaban	Apixaban	Edoxaban
Incidence of menorrhagia^24^	4.5–9.6%	--	4.7%	9.5%	5.4%	9.0%
Relative risk of menorrhagia^24^	Reference	--	0.53 (*p*<0.01)	2.10 (*p*<0.01)	1.18	1.26
Crosses placenta^12^	Yes	No	Yes	Yes	Unknown	Unknown
Pregnancy risk^12^	High	Minimal	Moderate	Moderate	Moderate	Moderate
Lactation risk^13^	Minimal	Minimal	Moderate	Moderate	Unknown	Unknown

AUB can negatively impact many dimensions of a woman’s life and lead to self-discontinuation or dose reduction around menses, which can increase risk for recurrent thrombosis.^27,28^ Accordingly, clinicians caring for women of reproductive age on anticoagulation should obtain detailed menstrual histories prior to initiating anticoagulation and at least annually during treatment. Menstruating women should be screened with a complete blood count and ferritin at least annually. Clinicians should provide patients with anticipatory guidance and recommend management strategies for AUB, which can usually be effectively managed with hormonal medications.^19^

### Controlling Heavy Menses

Menstrual flow can be safely reduced with several medications that do not adversely impact future fertility. Progestin-only preparations may be preferable over estrogen-containing preparations for patients on anticoagulation, as current evidence suggests they do not increase thrombosis risk.^29–31^ The only contraindication to use of progestin-only formulations is a personal history of breast cancer.^29^ Progestin-only products include pills (norethindrone 0.35 mg (sometimes called the “mini-pill”), drosperinone 4 mg, norethindrone 5–10mg, and outside of the US, desogestrel 75mcg), depot medroxyprogesterone acetate (DMPA) injections, levonorgestrel-containing intrauterine devices (IUD), and the etonogestrel subdermal arm implant (Nexplanon). Among these options, the 52 mg levonorgesterel-bearing IUD (marketed as Liletta and Mirena) is the most effective in controlling menses among women with AUB.^32^ An IUD can be safely placed during a simple office visit for women who are anticoagulated.^33,34^ Lower dose levonorgestrel-containing IUDs (Skyla or Kyleena) are safe but show no advantage over the higher dose IUDs and are less effective at controlling menses.

The use of combined estrogen-progestin medications by patients at increased risk of thrombosis is controversial.^35^ Estrogen has been shown to have a dose-dependent thrombotic risk, with the highest thrombotic risk in patients treated with 50μg ethinylestradiol-levonorgestrel and the lowest in 20–30μg ethinylestradiol-levonorgestrel.^36^ A Cochrane meta-analysis on venous thromboembolism (VTE) risk associated with different combined oral contraceptives among women who were not anticoagulated found that when compared to levonorgestrel, other progestins’ pooled relative risk (95% confidence interval, CI) of VTE was 1.33 (1.08–1.63) for gestodene, 1.93 (1.31–2.83) for desogestrel, and and 1.67 (1.10–2.55) for drospirenone.^37^ A prior systematic review also found that levonorgestrel was associated with lower thrombotic risk compared to other progestins: relative risk of VTE (95% CI) of ethinylestradiol with progestins in patients taking combined oral contraceptives versus non-users was 2.9 (2.2–3.8) for levonorgestrel, 6.6 (5.6–7.8) for desogestrel, and 6.4 (5.4–7.5) for drospirenone.^38^

Tranexamic acid is another option for managing heavy menses, which has been available without a prescription in Scandinavia for decades. For women at risk of recurrent thrombosis, however, the safety of this medication remains controversial. One large historical prospective cohort study showed an increased incidence of VTE in women of reproductive age treated with tranexamic acid (without anticoagulation) compared to those not treated, though this small increase in VTE risk is not clinically significant for use during menstruation alone (number needed to harm per 5 days of treatment was 78,549 persons).^39^ A meta-analysis of a more general, non-surgical patient population treated with tranexamic acid for indications including but not limited to menstrual bleeding demonstrated reduction in all-cause mortality without increased venous or arterial thrombotic complications.^40^ While further studies that control for indication for anticoagulation are needed, many clinicians cautiously recommend tranexamic acid to control troublesome menstrual bleeding in women treated with anticoagulation.^41^

Patients on anticoagulation should be also counseled to avoid frequent use of non-steroidal anti-inflammatory drugs (NSAIDs), which they may have previously used to control menstrual cramping, as NSAIDs increase the possibility of bleeding events and gastrointestinal irritation. In the Randomized Evaluation of Long Term Anticoagulant Therapy trial, a considerable proportion (12.6%, *n*=2,279) of patients on either warfarin or dabigatran used NSAIDs at least once during the trial.^42^ NSAID use by these patients significantly elevated the rate of major bleeding (hazard ratio (HR): 1.68; 95% CI 1.40–2.02; *p*<0.0001), gastrointestinal major bleed (HR: 1.81; 95% CI 1.35–2.43; *p*<0.0001), stroke or systemic embolism (HR: 1.50; 95% CI: 1.12–2.01; *p*=0.007), and hospitalization frequency (HR 1.64; 95% CI 1.51–1.77; *p*<0.0001). For this reason, patients requiring anticoagulation should be cautioned not to use more than a single dose of NSAIDs.

When medications are not effective in controlling troublesome menstrual flow, a number of gynecologic procedures may be considered. These include endometrial ablation, uterine artery embolization or myomectomy to address leiomyomas, or hysterectomy. However, these procedures may be inappropriate if future fertility is desired.^43^

### Prevention of Hemorrhagic Ovarian Cysts

Studies show that 4% of women will be admitted to the hospital for an ovarian cyst by the age of 65, with this risk increased by inherited thrombophilia and anticoagulation use.^3^ Unfortunately, hemorrhagic ovarian cysts carry significant risk of morbidity and mortality. Hemorrhagic ovarian cysts occur more frequently in women on anticoagulation, and particularly in those who are supratherapeutic (i.e., international normalized ratio or INR greater than 4).^4^ Hormonal treatments which suppress ovulation are effective in preventing the formation of ovarian cysts. These include progestin-only methods, such as the etonogestrel implant (Nexplanon), DMPA, and combined estrogen and progestin hormonal pills, patch, or ring. The levonorgestrel IUD and progestin-only pills suppress ovulation for some but not all women and are therefore not preferred for hemorrhagic ovarian cyst prevention.^44,45^ Given its superior contraceptive effectiveness and ability to reliably reduce risk of hemorrhagic ovarian cysts, the subdermal implant may be considered a first-line option for those requiring anticoagulation.

## PREGNANCY CONSIDERATIONS FOR WOMEN ON ANTICOAGULATION

Women currently on anticoagulation, as well as those with a history of VTE who desire pregnancy, should establish care with an obstetrician or a maternal fetal medicine specialist prior to becoming pregnant. There is no evidence that anticoagulant medications, including warfarin, enoxaparin, and DOACs, limit fertility. However, there are risks, such as embryopathy, fetal hemorrhage, and obstetric bleeding, associated with use of certain anticoagulants during pregnancy. Table 2 compares the known teratogenic risk of common anticoagulants.^12^

Warfarin is a known teratogen. It has been demonstrated to cross the placenta and result in major congenital malformations, fetal hemorrhage in utero, and increased risk of spontaneous abortion or fetal mortality. For women treated with warfarin who are planning pregnancy, one approach to minimize the risk of warfarin-associated embryopathy suggested by the 2012 American College of Chest Physicians Guidelines is to continue warfarin, frequently test for pregnancy, and transition to low molecular weight heparin (LMWH) as soon as pregnancy is established.^46^ To effectively use this approach, women must have regular menstrual cycles and agree to frequent pregnancy testing. Alternatively, warfarin may be replaced by LMWH prior to attempting conception.^47^ LMWH does not cross the placenta and is the current anticoagulant of choice for pregnant women requiring thrombosis prophylaxis or treatment.^46,48^

There is less evidence regarding the potential teratogenicity of DOACs. Rivaroxaban and dabigatran appear to cross the placenta, but it is unknown whether apixaban or edoxaban do. One case report on the inadvertent use of rivaroxaban throughout pregnancy showed no evidence of teratogenic effects.^49^ Physician reporting on inadvertent DOAC exposure during pregnancy has showed that among 137 cases, 7 showed fetal abnormalities (5.1%), of which 3 (2.2%) were consistent with embryopathy.^50^ Another case series involving 63 pregnancies with rivaroxaban exposure found only one malformation (conotruncal cardiac defect) which occurred for a woman who had previously had a pregnancy affected by a fetal cardiac malformation without rivaroxaban exposure.^51^ According to this limited data, pregnant women are advised to avoid use of DOACs during pregnancy.^46^

### Prevention of Unwanted Pregnancy

Given the risks of anticoagulation during pregnancy, clinicians should routinely discuss patients’ sexual practices and reproductive goals, as well as assess potential for pregnancy prior to and during anticoagulant use. This includes educating patients regarding available contraceptive methods, counseling on non-contraceptive benefits of contraceptive medications regardless of pregnancy risk, assessing adherence to chosen contraceptive method, and consideration of pregnancy testing. Tables 3 and 4 compare the many options available to prevent unwanted pregnancy which should be discussed with patients prior to starting anticoagulation. Detailed information regarding Medical Eligibility for Contraception Use has been compiled by the US Centers for Disease Control^52^ and the World Health Organization^53^ and is freely available to clinicians online.

**Table 3:** Family Planning Method Effectiveness and Impact on Menstrual Flow and Ovulatory Suppression

Contraceptive method	Unintended pregnancy in first year with typical use*^54,55,57,58^	Women continuing contraceptive use after one year^52,56^	Menstrual flow	Ovulatory suppression
Etonogestrel implant (Nexplanon)	0.05%	83–84%	Reduce	Yes
Vasectomy (male)	0.15%	Permanent	No effect	No
Levonorgestrel IUD (Liletta, Mirena)	0.2–2.4%	80–88%	Reduce	Variable
Tubal ligation	0.5–2.6%	Permanent	No effect	No
Copper IUD (Paragard)	0.8–3.0%	78–84%	Increase	No
DMPA injection (Depo-Provera)	3–6%	56%	Reduce	Yes
Combined estrogen-progestin pills, patch, ring	7–9%	55–68%	Reduce	Yes
Progestin-only pills			Reduce	Variable
Condoms, male	12–18%	68%	No effect	No
Condoms, female	21%	68%	No effect	No
No method	85%	n/a	No effect	No

*Pregnancy rates among typical couples who initiate use of a method and do not stop use

**Table 4:** Emergency Contraception Effectiveness and Treatment-Associated Bleeding Without Anticoagulation

Emergency contraceptive methods^62,64^	Observed number pregnancies (95% CI)	Relative risk (95% CI)	Bleeding after treatment (95% CI)	Relative risk (95% CI)
Mifepristone 25–50mg^†^ vs Levonorgestrel 1.5mg	21 per 1000 (16 to 29) 35 per 1000	RR 0.61 (0.45 to 0.83)	47 per 1000 (32 to 68) 77 per 1000	RR 0.61 (0.42 to 0.88)
Ulipristal acetate vs Levonorgestrel (all doses)	13 per 1000 (8 to 22) 22 per 1000	RR 0.59 (0.35 to 0.99)	6 per 1000 (2 to 20) 9 per 1000	RR 0.71 (0.23 to 2.24)
Copper IUD vs Mifepristone (all doses)^†^	4 per 1000 12 per 1000 (0–34)	RR 0.33 (0.04 to 2.74)	Not reported Not reported	
Copper IUD vs Levonorgestrel IUD	0 per 1000 (0–10) 3 per 1000 (0–17)		18 per 1000 15 per 1000	Not reported

^†^Low-dose mifepristone is available in many countries, though only higher dose formulations are available in the USA

The most effective contraceptives are the etonogestrel implant (Nexplanon) and 52mg levonorgestrel-containing IUD (Liletta or Mirena).^54^ These contraceptives are rapidly reversible when pregnancy is desired and typically more effective than permanent contraception with tubal ligation.^55^ Further, the subdermal implant and IUDs have very high rates of user satisfaction and continuation.^56^ Although the contraceptive pill, patch, and ring are effective methods of contraception, they require reliable patient adherence to be effective. Since it is difficult for some patients to remember to take a pill every day, pregnancy rates with typical use of oral contraceptives are estimated at 8–9% within the first year of use.^57,58^ Among progestin-only contraceptives, the 0.35mg norethindrone-only pill is the most sensitive to missed or late doses; the newest drosperinone-only pill (Slynd) has a longer half-life and so is less sensitive to timing of doses.^59^ The use of barrier methods (i.e., condoms, diaphragms) without another form of contraception can result in failure rates that some patients consider unacceptably high, particularly when treated with a teratogenic medication.^60^ When a patient prefers to rely on a barrier or behavioral method, such as withdrawal, they should be provided with information about and a prescription for emergency contraceptive pills. Depending on gestational age and individual risk factors, termination of an undesired pregnancy (whether with a medication or procedure) can usually be safely performed without interrupting anticoagulation.^61^

Emergency contraceptive (EC) pills can be safely used by women treated with anticoagulation who wish to avoid pregnancy after unprotected sex.^29^ Levonorgestrel EC (Plan B One-Step, Next Choice) is available without a prescription to those over 16 years of age in the USA. It is more effective the sooner it is taken after unprotected or inadequately protected intercourse, though pills can be used up to 5 days after a contraceptive emergency. Ulipristal acetate is an alternative oral EC agent available by prescription in the USA that is typically twice as effective as levonorgestrel EC^62^; it may be more effective for obese women than levonorgestrel pills, though effectiveness of both agents appears to be reduced by obesity.^56^ Low-dose mifepristone is another EC pill used in many countries, though only higher dose formulations used in medical abortion are available in the USA. Patients who do not use highly effective contraception should be advised to maintain a supply of EC pills on hand for immediate use in case a contraceptive emergency arises. EC pill effectiveness may be reduced by obesity, delays in treatment beyond 72 h, repeated acts of unprotected intercourse, and unprotected intercourse around the time of ovulation.^63^ In addition to these oral medications, placement of a levonorgestrel or copper-bearing IUD within 5 days of unprotected intercourse is highly effective as EC.^62,64^ As copper IUDs may increase menstrual flow, they should be used with caution in women treated with anticoagulation.^29^ Table 4 summarizes the effectiveness and treatment-associated bleeding of these EC agents.

## BREASTFEEDING WHILE ON ANTICOAGULATION

Breastfeeding is the preferred means of providing nutrition to newborns and has long-term health benefits for mothers.^65^ Outcomes with breastfeeding while on anticoagulation are well studied in warfarin and enoxaparin; less evidence is available for breastfeeding outcomes while on DOACs. Informed by current understanding of pharmacokinetics, Table 2 summarizes available information on the use of six common anticoagulants during breastfeeding.^13^

Warfarin has demonstrated very low levels in breastmilk and no adverse reactions in infants breastfed by mothers treated with warfarin, even with doses up to 25mg daily for 7 days. Several organizations, including the American Academy of Pediatrics and American College of Chest Physicians,^46,66^ recommend no change in warfarin therapy during breastfeeding. While less data are available for enoxaparin, the medication’s high molecular weight is unlikely excreted in breastmilk nor absorbed by breastfed infants and there have been no reports of adverse effects. Thus, no special precautions are recommended for breastfeeding mothers treated with warfarin or enoxaparin.

Apixaban is not recommended for use during breastfeeding because high levels have been demonstrated in breastmilk. In contrast, rivaroxaban has been identified at only low levels in breastmilk from mothers treated with 15 to 30mg daily, without reports of adverse effects on infants. Dabigatran similarly appears to be poorly excreted into breastmilk and is unlikely to affect a breastfed infant. Although the American College of Chest Physicians recommends avoiding DOACs during breastfeeding,^46^ rivaroxaban and dabigatran have been used by breastfeeding mothers, with careful monitoring of infants for any signs of bleeding.^67^ There are fewer data available on edoxaban use during breastfeeding, so it is not currently recommended. To better understand the risks associated with use of these medications, the International Society on Thrombosis and Haemostasis has a repository for clinicians to report cases of DOAC exposure during pregnancy and lactation.^68^

## CONCLUSION

There are currently multiple medications available for patients who require anticoagulation and many options for helping young women avoid menorrhagia and undesired pregnancy. Choice of anticoagulant, as well as management of complications such as bleeding and recurrent thrombosis, will vary based on indication for anticoagulation. Nonetheless, general internists are well suited to involve patients in anticoagulation management, in collaboration with their hematology, gynecology, cardiology, rheumatology, and/or pulmonary medicine colleagues. In addition to clinical and lifestyle factors, discussion of finances, formularies, and insurance is also often required.

Research is needed to understand the multiple dimensions of how anticoagulation impacts patients’ quality of life. For example, studies are ongoing to evaluate the impact of DOACs on AUB; the RAMBLE (Rivaroxaban vs Apixaban for Heavy Menstrual Bleeding) and MEDEA (Heavy MEnstrual bleeding in premenopausal women treated with DirEct oral Anticoagulants) trials should contribute important information.^25,69^ Studies are also needed on the effects of dual antiplatelet therapy for women of reproductive age.^70^ Based on available data, clinicians are most likely to help young women avoid thrombotic complications by routinely assessing menstrual symptoms and reproductive goals, and engaging patients in effective shared decision-making regarding anticoagulation.

### Text Box. Key Summary Points

**Table:** 

• Clinicians should review patients’ menstrual histories prior to initiating anticoagulation and regularly during treatment.	
• Clinicians should routinely assess anticoagulant adherence, offer assistance controlling heavy menses, and caution patients not to discontinue or dose-reduce anticoagulant use.	
• Clinicians should routinely assess potential for unwanted pregnancy and counsel patients on the comparative effectiveness of available contraceptive methods.	

## Supplementary Information

ESM 1 (DOCX 45 kb)
