# Supplementary material for: Special Considerations for Women of Reproductive Age on Anticoagulation
Source: J Gen Intern Med. 2022 May 31;37(11):2803–10. doi: 10.1007/s11606-022-07528-y (PMC9411301; doi:10.1007/s11606-022-07528-y)
Supplement: Supplementary file 1 — (DOCX 45 kb) [file 11606_2022_7528_MOESM1_ESM.docx]

**Supplementary Figure. Review of abnormal uterine bleeding experienced by women of reproductive age on anticoagulation**

**Identification of studies via database search**

Records removed *before screening*:

Duplicate records removed (n = 8)

Non-English language (n = 1)

Records identified from:

Databases (n = 2)

PubMed

EMBASE

**Identification**

Records excluded if related to (n = 170):

Pregnancy and obstetric complications (n = 38)

Cardiac and vascular conditions (n = 26)

Rheumatologic conditions (n = 20)

Hematologic conditions (n = 12)

Malignancy (n = 8)

Cerebral Vein Thrombosis (n = 8)

Other (n = 58)

Records screened (n = 204)

PubMed (n = 107)

EMBASE (n = 97)

Reports unable to be retrieved (n = 2)

Reports sought for retrieval

(n = 30)

**Screening**

Reports excluded if related to (n = 8):

Different population studied (n = 4)

Similar article by same author retrieved (n = 2)

Beyond scope of this review (n = 2)

Reports assessed for eligibility

(n = 28)

Studies included (n = 20)

PubMed (n = 12/15)

EMBASE (n = 8/13)

**Included**
